# Mapping the Potential Geographic Distribution of the Heartwater Disease Vector Tick *Amblyomma hebraeum* Under Climate Change

**DOI:** 10.3390/ani16101455

**Published:** 2026-05-09

**Authors:** Mohammed Okely, Areej A. Al-Khalaf, Mohamed G. Nasser, Abdelwahab Khalil, Sara A. AlAshaal

**Affiliations:** 1 Entomology Department, Faculty of Science, Ain Shams University, Abbassia, Cairo 11566, Egypt; 2 Biology Department, College of Science, Princess Nourah Bint Abdulrahman University, Riyadh 11671, Saudi Arabia; aaalkhalaf@pnu.edu.sa; 3 Research Lab of Biogeography and Wildlife Parasitology, Entomology Department, Faculty of Science, Ain Shams University, Abbassia, Cairo 11566, Egypt; medo.entomophelia@gmail.com (M.G.N.); alashalsara@gmail.com (S.A.A.); 4 Entomology Division, Zoology Department, Faculty of Science, Beni-Suef University, Beni-Suef 62521, Egypt; abdelwahab.khalil@science.bsu.edu.eg

**Keywords:** *Amblyomma hebraeum*, niche modeling, MaxEnt, suitable habitat, risk maps, climate change

## Abstract

*Amblyomma hebraeum* is a tick species of veterinary importance because it is a major vector of *Ehrlichia ruminantium*, the causative agent of heartwater disease. This species infests livestock in southern African regions and is a three-host species of veterinary importance. Maximum entropy algorithm (MaxEnt) modeling was used to predict the potential distribution of *A. hebraeum*. The MaxEnt model performed better than random with an average test area under the curve (AUC) value of 0.94, and model predictions were significantly better than random, giving AUC ratios above the null expectations in the partial receiver operating characteristic (pROC) analyses (*p* < 0.001). A set of five variables was selected for the species from 19 bioclimatic variables based on correlation analysis. The study showed that the current distribution of *A. hebraeum* is estimated to occur across coastal regions of the Eastern Cape and KwaZulu-Natal provinces in South Africa, Swaziland, southern Mozambique, eastern Zimbabwe, and eastern Botswana. Temperature seasonality (Bio 4) had the highest effect on the distribution of this species. The environmentally suitable habitat for *A. hebraeum* increased with decreasing temperature seasonality (Bio 4). Transferring the models to future conditions showed a decrease in suitable habitats for this species under changing climate. These results have public health implications and can be used for making control planning decisions in areas suitable for this vector across its geographical distribution.

## Simple Summary

Ticks are small parasites that feed on animals and can spread dangerous diseases. *Amblyomma hebraeum*, known as the South African bont tick, is particularly important because it transmits heartwater disease to cattle, sheep, and goats, causing significant economic losses to livestock farmers across southern Africa. This tick also carries diseases that can infect humans. Climate plays a crucial role in determining where ticks can survive. We used computer modeling to map where this tick species currently lives and predict how climate change might affect its distribution in the future. We analyzed 734 location records from across southern Africa and examined 19 different climate factors, ultimately identifying five key variables that determine where the tick can survive: temperature stability throughout the year, seasonal temperature variation, winter temperatures, annual rainfall, and peak monthly rainfall. Our results show that this tick prefers areas with stable temperatures and moderate rainfall, which explains why it is most common along South Africa’s eastern coast. Concerningly, our predictions suggest that climate change will reduce suitable habitat for this tick by 5% to 27% by the year 2100, depending on how much global temperatures rise. However, the tick will not disappear entirely—it will remain in core areas while losing habitat in marginal regions. These findings help veterinarians, farmers, and public health officials understand where tick-borne diseases pose the greatest risk now and in the future, allowing them to focus disease prevention efforts where they are needed most as our climate continues to change.

## 1. Introduction

The South African bont tick, *Amblyomma hebraeum* Koch, 1844, represents a critical arthropod vector of veterinary and medical importance throughout southern Africa. This three-host tick species exhibits a broad host range across multiple developmental stages [1,2]. Adult ticks commonly infest large wild ungulates such as giraffes, buffaloes, elands, and rhinoceroses. In addition to these native hosts, they also parasitize domestic livestock, including cattle, sheep, and goats. The immature stages (larvae and nymphs) exhibit similarly broad host preferences, feeding on both large wild mammals and smaller vertebrates such as antelopes, scrub hares, game birds, helmeted guineafowls, and tortoises [3,4]. This remarkable host plasticity contributes to the species’ ecological success and underscores its significance as a disease vector.

The veterinary and public health importance of *A. hebraeum* stems primarily from its capacity to transmit multiple pathogens of economic and medical concern. Most notably, this tick serves as the principal vector of *Ehrlichia ruminantium*, the causative agent of heartwater disease, which poses a substantial threat to livestock productivity across its range. Beyond veterinary pathogens, *A. hebraeum* also transmits agents of human disease, including *Rickettsia africae* and *Rickettsia conorii*, both of which cause tick-borne rickettsioses in humans. Additionally, this species transmits *Theileria mutans* and *Theileria velifera*, parasites responsible for benign theileriosis in cattle [1,3].

*Amblyomma hebraeum* is strongly associated with warm environments that combine adequate moisture with structured vegetation, particularly savanna and bushveld ecosystems [1]. The species depends on vegetated habitats that support both microclimatic stability and host availability, while its absence from open grasslands highlights the importance of vegetation cover as a key ecological constraint shaping its realized distribution [3,5].

The geographic range of *A. hebraeum* extends across substantial portions of southern Africa, though its distribution is far from uniform. Areas of particularly high abundance include the coastal regions of the Eastern Cape and KwaZulu-Natal provinces in South Africa, as well as Swaziland, southern Mozambique, southern and eastern regions of Zimbabwe, and eastern Botswana [1,3]. Beyond these core areas, populations have been documented in Namibia [1], the Muheza District of Tanzania [6], and portions of eastern Kenya [7]. This fragmented distribution pattern reflects the species’ specific climatic requirements and habitat preferences. In addition to climatic factors, biotic interactions may also play a role in shaping the realized distribution of *A. hebraeum*. In particular, overlap with other tick species such as *A. variegatum* may influence local distribution patterns. Previous studies have documented parapatric boundaries between these two species, suggesting that competitive or reproductive interactions may contribute to defining their distribution limits [8].

In recent years, ecological niche modeling has emerged as a powerful tool for understanding and predicting the spatial distribution of disease vectors under both current and future environmental conditions. This approach has been successfully applied to numerous tick species globally, providing critical insights for disease surveillance and control planning [9,10,11,12,13,14,15,16]. Despite this growing body of literature, comprehensive assessments of climate change impacts on *A. hebraeum* across its entire geographic range remain limited.

Previous research on the potential distribution of *A. hebraeum* has been largely confined to local or regional scales. In Zimbabwe, two studies have examined the current and projected distribution of this species under climate change scenarios [17,18]. Similarly, Estrada-Peña [19] investigated the potential effects of climate warming on *A. hebraeum* distribution within South Africa. While previous efforts have modeled the distribution of *A. hebraeum*, several limitations remain. Earlier studies primarily relied on old versions and coarse climatic variables and, in some cases, restricted their occurrence records to specific host species, with limited consideration of sampling bias, multicollinearity among predictors, and model interpretability. In addition, comprehensive assessments of future climate change impacts on this species remain limited. Furthermore, none of these investigations assessed the species’ potential distribution across its complete geographic range in southern Africa.

The present study addresses these knowledge gaps by providing a comprehensive assessment of climate change effects on the potential distribution of *A. hebraeum* throughout its entire southern African range, integrating a large, rigorously curated occurrence dataset, applying spatial filtering to reduce sampling bias, and implementing a robust variable selection framework combining correlation analysis, Variance Inflation Factor (VIF), and ecological relevance. We employed the maximum entropy algorithm (MaxEnt) for ecological niche modeling, utilizing the most current climate data (WorldClim 2.1) and multiple future climate scenarios. By incorporating occurrence records from diverse host species obtained from biodiversity databases and published literature, we aimed to capture the full ecological breadth of this tick species. Our objectives were threefold: (1) to model the current potential distribution of *A. hebraeum* across southern Africa, (2) to project changes in habitat suitability under four future climate scenarios spanning 2041–2100, and (3) to identify the key environmental factors limiting the species’ distribution. The resulting maps and analyses provide an improved foundation for understanding the ecology of this important disease vector and support evidence-based control planning in an era of accelerating environmental change.

## 2. Materials and Methods

### 2.1. Occurrence Records

Occurrence data for *A. hebraeum* were compiled from multiple sources to ensure comprehensive geographic coverage. Primary data sources included the VectorMap database (www.vectormap.org), a specialized repository for arthropod vector distribution data, and iNaturalist (www.inaturalist.org), a citizen science platform providing geo-referenced biodiversity observations. These digital repositories were supplemented with occurrence records extracted from peer-reviewed literature, specifically from studies documenting tick distributions across southern Africa [6,7,20,21,22]. We downloaded an initial set of 807 occurrence records for *A. hebraeum*. To maintain data quality and spatial precision, only records accompanied by geographic coordinates were retained for subsequent analyses.

The compiled dataset underwent rigorous quality control procedures to address common issues in species distribution modeling. Duplicate records—those representing identical geographic locations from multiple sources—were identified and removed to prevent spatial autocorrelation and overfitting of the model. After this step, the data yielded 778 unique records for *A. hebraeum.* To further minimize sampling bias and spatial clustering, we employed the spatially rarefy function implemented in SDMtoolbox 2.4 [23] within ArcGIS 10.3 (Environmental Systems Research Institute, Redlands, CA, USA). This procedure applies a distance filter to eliminate redundant occurrences within individual grid cells, with a threshold set at 2.5 arcminutes (approximately 5 km at the equator) to match the resolution of the environmental predictors (WorldClim v2.1), ensuring spatial consistency between occurrence records and environmental layers. This step reduces spatial autocorrelation and sampling bias while maintaining ecologically meaningful environmental variation across the study area [24,25]. Finally, occurrence records for this vector species were reduced to 734 spatially unique points (Supplementary File). Following these filtering steps, the final occurrence dataset was randomly partitioned into two equal subsets: 50% for model calibration (training) and 50% for independent model evaluation (testing), following established best practices in ecological niche modeling [26].

### 2.2. Environmental Variables and Data Extraction

Climatic data were obtained from the WorldClim version 2.1 database (www.worldclim.org), which provides high-resolution climate surfaces for global land areas [27]. This version represents a substantial improvement over previous iterations in terms of spatial accuracy and temporal coverage, making it particularly suitable for species distribution modeling applications. All climate layers were downloaded at a spatial resolution of 2.5 arcminutes (approximately 4.5 km at the equator) for both current conditions (averaged over the period 1970–2000) and future climate projections. The WorldClim database provides 19 bioclimatic variables (BIO1–BIO19) derived from monthly temperature and precipitation values. These variables capture ecologically meaningful aspects of climate, including annual trends, seasonality, and extreme environmental conditions [27]. However, following recommendations from recent methodological studies [28,29], we excluded four variables (BIO8, BIO9, BIO18, and BIO19) from our initial variable pool due to documented spatial artifacts and discontinuities at the edges of climate zones, which can produce unreliable predictions in species distribution models.

For each georeferenced occurrence point, we extracted the corresponding values of the 15 remaining bioclimatic variables using DIVA-GIS version 7.5 [30], a widely used geographic information system for biodiversity analysis. This extraction procedure assigns the environmental values from each climate layer to the precise coordinates of each occurrence record, creating a matrix of climate values associated with the known distribution of *A. hebraeum*. These extracted values formed the basis for subsequent variable selection and model calibration procedures.

### 2.3. Variable Selection and Multicollinearity Analysis

Multicollinearity among environmental predictors represents a well-documented challenge in species distribution modeling, as highly correlated variables can lead to model overfitting, inflated parameter estimates, and reduced transferability to new geographic areas or time periods [31,32]. To address this issue, we implemented a multi-step variable selection protocol combining correlation analysis, principal component analysis, and ecological interpretation.

First, we conducted a comprehensive pairwise correlation analysis of all 15 retained bioclimatic variables using Pearson’s correlation coefficient. This analysis was performed in JMP Pro 16 (SAS Institute Inc., Cary, NC, USA), utilizing the multivariate analysis platform to generate a complete correlation matrix. We applied a conservative threshold of |r| > 0.7 to identify problematic correlations, following widely adopted guidelines for addressing multicollinearity in ecological modeling [31]. When pairs of variables exceeded this threshold, we retained the variable with greater ecological relevance to tick biology based on published literature, or the variable showing stronger univariate relationships with species presence in preliminary analyses.

To complement the correlation analysis and gain additional insights into the structure of environmental variation across the study region, we performed a principal component analysis (PCA) on the full set of 15 bioclimatic variables. This analysis was conducted in JMP Pro 16 using the standardized variables (mean-centered and scaled to unit variance) to account for differences in measurement units among variables. The PCA provided eigenvalues, eigenvectors, and variable loadings that helped identify the major axes of climatic variation and confirmed patterns revealed by the correlation analysis [33]. Variables with high loadings on the same principal components were considered potentially redundant, providing an independent validation of the correlation-based selection. Principal Component Analysis (PCA) was used only as an exploratory tool to visualize the environmental structure of the dataset and to assist in variable selection. The final model was developed using the original bioclimatic variables to preserve ecological interpretability. Additionally, we used the jackknife procedure implemented in MaxEnt to evaluate the relative importance of each variable for predicting species distribution. To refine variable selection, we calculated the Variance Inflation Factor (VIF) using the usdm package in R [34,35] and removed variables with high VIF values. VIF values were interpreted following standard thresholds, with values below 5 indicating low multicollinearity among predictors [35].

This resampling approach systematically excludes each variable in turn and measures the resulting decrease in model performance, thereby quantifying each variable’s unique contribution to predictive accuracy [36]. The final selection of variables balanced four criteria: (1) low intercorrelation (|r| < 0.7), (2) strong individual predictive power based on jackknife analysis, (3) low Variance Inflation Factor, and (4) ecological interpretability in the context of tick biology and life history requirements.

Following this comprehensive selection process, five bioclimatic variables were retained for final model construction: Isothermality (BIO3), Temperature Seasonality (BIO4), Mean Temperature of Coldest Quarter (BIO11), Annual Precipitation (BIO12), and Precipitation of Wettest Month (BIO13). These variables capture key aspects of both thermal regime and moisture availability that are known to influence tick survival, development rates, questing behavior, and seasonal activity patterns [37,38].

### 2.4. Future Climate Scenarios

To project the potential impacts of climate change on *A. hebraeum* distribution, we obtained future climate projections from the WorldClim 2.1 database based on the Coupled Model Intercomparison Project Phase 6 (CMIP6) [39]. We selected four Global Climate Models (GCMs) that represent diverse climate sensitivities and have demonstrated good performance in simulating African climate patterns: the Beijing Climate Center Climate System Model (BCC-CSM2-MR), the Centre National de Recherches Météorologiques Earth System Model (CNRM-ESM2-1), the Model for Interdisciplinary Research on Climate version 6 (MIROC6), and the MIROC Earth System version 2 for Long-term simulations (MIROC-ES2L) [40].

For each GCM, we obtained projections under four Shared Socioeconomic Pathways (SSPs): SSP1-2.6 (low emissions, sustainable development), SSP2-4.5 (intermediate emissions, middle-of-the-road development), SSP3-7.0 (high emissions, regional rivalry), and SSP5-8.5 (very high emissions, fossil-fueled development) [41]. These scenarios span a comprehensive range of possible future radiative forcing levels and socioeconomic trajectories. Projections were analyzed for three future time periods: 2041–2060 (near-term), 2061–2080 (mid-term), and 2081–2100 (long-term), providing insights into both imminent changes and longer-term trends.

To reduce uncertainty associated with reliance on a single climate model and produce more robust predictions, we calculated ensemble forecasts by averaging the predictions from all four GCMs for each SSP and time period [42]. This ensemble approach has been shown to outperform individual models by compensating for model-specific biases and uncertainties [43]. The study area was extracted from the global environmental layers using the “Extract by Mask” tool in ArcGIS 10.3, and all layers were saved in ASCII grid format to meet MaxEnt input requirements [44,45].

### 2.5. Ecological Niche Modeling

Species distribution models were constructed using the maximum entropy approach as implemented in MaxEnt version 3.4.4 [46]. MaxEnt has become one of the most widely used algorithms for ecological niche modeling due to several methodological advantages: it performs well with presence-only occurrence data (which are more readily available than presence-absence data for most species), demonstrates robust performance with small sample sizes, handles complex interactions between environmental variables, and has consistently shown high predictive accuracy in comparative studies [47,48].

The algorithm generates a probability distribution of habitat suitability ranging from 0 (lowest suitability) to 1 (highest suitability) by finding the distribution of maximum entropy (i.e., closest to uniform) subject to the constraint that the expected value of each environmental variable under this distribution matches its empirical average calculated from the occurrence data [46]. We configured MaxEnt to use auto-features, which automatically selects appropriate combinations of feature types (linear, quadratic, product, threshold, and hinge) based on the number of occurrence records, thereby optimizing model complexity relative to sample size [49].

To ensure robust parameter estimation and account for stochasticity in the modeling process, we ran 10 replicate models using bootstrap resampling with a random seed, and the final predictions represent the mean across all replicates [50]. The maximum number of iterations was set to 5000 to ensure model convergence, and we used a regularization multiplier of 1.0 (the default value) to balance model fit and complexity. To avoid unreliable extrapolation when projecting models to future climate scenarios—where environmental conditions may fall outside the range of values present in the training data—we disabled both the clamping and extrapolation options in MaxEnt [51,52]. This conservative approach restricts predictions to the environmental space represented in the calibration data, and avoids generating predictions under environmental conditions that fall outside the range of the calibration data, which may lead to biologically unrealistic output [53,54]. MaxEnt automatically generates response curves illustrating the relationship between habitat suitability and each environmental variable while holding other variables at their average values. These curves provide important insights into the species’ environmental tolerances and preferences [36]. Additionally, MaxEnt produces variable importance metrics through permutation importance and percent contribution statistics, which quantify the relative influence of each predictor on model performance.

### 2.6. Model Evaluation and Validation

Model performance was evaluated using multiple complementary approaches to provide a comprehensive assessment of predictive accuracy. First, we calculated the area under the receiver operating characteristic curve (AUC), a threshold-independent metric that measures the model’s ability to discriminate between presence and background localities [55]. AUC values range from 0.5 (performance no better than random) to 1.0 (perfect discrimination), with conventional interpretation thresholds as follows: poor (0.5–0.6), fair (0.6–0.7), good (0.7–0.8), very good (0.8–0.9), and excellent (0.9–1.0) [56]. We calculated AUC values for both the training dataset and the independent test dataset, with the latter providing a more rigorous assessment of model transferability.

Because standard AUC has been criticized for potentially inflating performance metrics when used with presence-only data and large study areas [57], we also implemented the partial ROC (pROC) test as an additional validation measure. This approach evaluates model performance across the range of omission errors that are most relevant for conservation and risk assessment applications, typically focusing on omission rates below 10% [58]. The pROC analysis was conducted using the NicheToolBox platform [59], with 500 bootstrap iterations and an E = 5% omission threshold to generate confidence intervals. Models were considered statistically significant when the AUC ratio (observed AUC/expected AUC under a null model) exceeded 1.0 at *p* < 0.05.

### 2.7. Niche Overlap and Limiting Factor Analyses

To quantify changes in habitat suitability between current and future climate scenarios, we calculated the total number of suitable grid cells (defined as pixels with suitability values ≥ 0.5) using the “Project Raster” and “Raster Calculator” functions in ArcGIS 10.3. This threshold-based approach allowed us to estimate proportional changes in potentially suitable habitat area under each future scenario and time period.

We performed niche overlap analysis using the envelope test function in DIVA-GIS 7.5 [30] to compare the environmental space occupied by *A. hebraeum* with the available environmental space across the study region. This analysis constructs multidimensional envelopes around occurrence points in environmental space and calculates the proportion of available habitat that falls within the species’ realized niche [60]. The envelope test provides insights into niche breadth and helps identify whether the species is a generalist or specialist with respect to the selected environmental variables.

To identify which environmental factors impose the strongest constraints on species distribution in different parts of the study area, we conducted a limiting factor analysis using the corresponding function in DIVA-GIS [30,61]. This analysis examines each grid cell across the study region and determines which environmental variable, when considered individually, would predict the lowest habitat suitability at that location. The resulting map indicates the primary limiting factor for each location, revealing spatial patterns in environmental constraints and highlighting areas where different climatic variables control distribution boundaries [62]. This information is particularly valuable for understanding mechanisms of climate change impacts and identifying regions where specific aspects of climate change (e.g., increased temperature seasonality versus altered precipitation patterns) are most likely to affect the species’ distribution.

## 3. Results

### 3.1. Occurrence Data and Spatial Distribution

**Figure 1:**
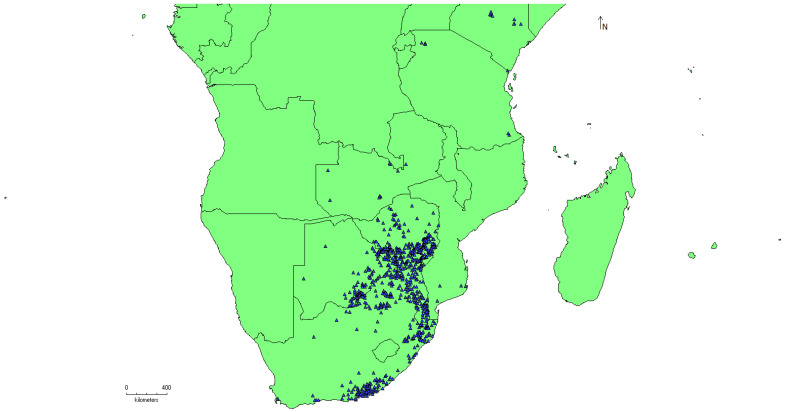
A total of 734 georeferenced occurrence records for *Amblyomma hebraeum* used for the modeling of this species using MaxEnt.

Following comprehensive data compilation and quality control procedures, a total of 734 georeferenced occurrence records for *Amblyomma hebraeum* were retained for analysis. These records encompassed the species’ known distribution across southern Africa, spanning multiple countries including South Africa, Mozambique, Zimbabwe, Botswana, Namibia, Tanzania, and Kenya. The spatial filtering process successfully reduced sampling bias while maintaining adequate geographic coverage across diverse environmental gradients, with occurrence points distributed across climatic zones ranging from humid coastal regions to semi-arid interior plateaus (Figure 1).

### 3.2. Variable Selection and Multicollinearity Assessment

**Figure 2:**
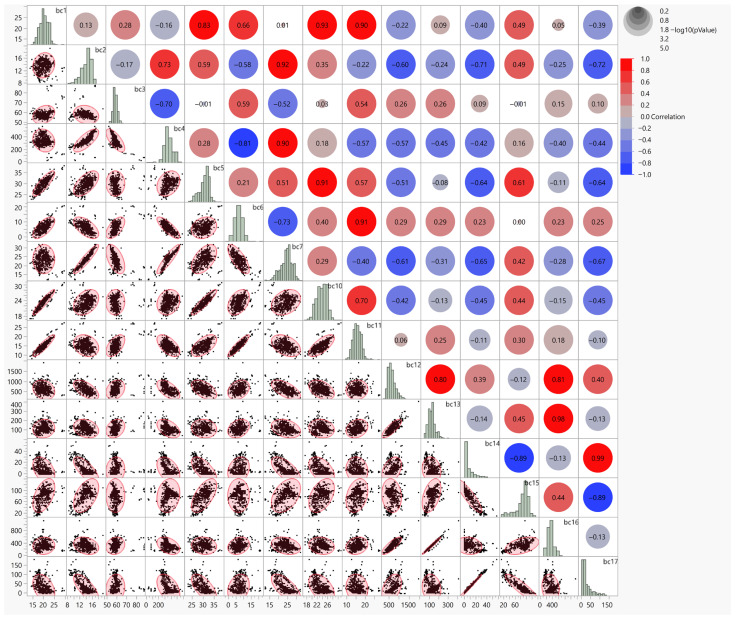
Correlation matrix and scatterplot array of bioclimatic variables used in this study.

The multivariate correlation analysis of the 19 bioclimatic variables revealed substantial intercorrelation among several predictors, underscoring the necessity for systematic variable selection to minimize multicollinearity effects in the distribution models (Figure 2). The correlation matrix demonstrated that numerous variable pairs exceeded the threshold of |r| > 0.7, with particularly strong correlations observed among temperature-related variables (BIO1, BIO5, BIO6, BIO10, BIO11) and among precipitation variables (BIO12, BIO13, BIO14, BIO16, BIO17). These patterns reflect the inherent covariation in climate systems, where variables derived from the same meteorological measurements naturally exhibit strong statistical dependencies.

**Figure 3:**
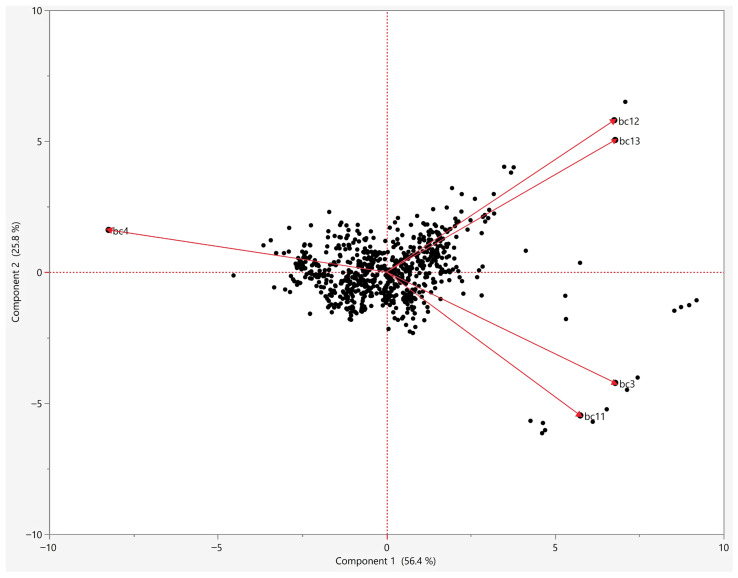
Principal component analysis (PCA) biplot of the 15 bioclimatic variables used in this study. Black points represent occurrence records, and red vectors indicate variable loadings.

Principal component analysis provided complementary insights into the structure of environmental variation across the study region (Figure 3). The first two principal components accounted for 56.4% and 25.8% of the total variance, respectively, together explaining 82.2% of the climatic variation. The biplot revealed distinct groupings of correlated variables, with precipitation variables (BIO12 and BIO13) loading strongly in the upper right quadrant, while temperature variables showed more dispersed patterns. Notably, BIO4 (Temperature Seasonality) occupied a relatively isolated position in the variable space, indicating its relative independence from other climatic predictors and supporting its retention as a key predictor variable. The combined evidence from correlation analysis, PCA, and jackknife testing guided the selection of five bioclimatic variables for final model construction: Isothermality (BIO3), Temperature Seasonality (BIO4), Mean Temperature of Coldest Quarter (BIO11), Annual Precipitation (BIO12), and Precipitation of Wettest Month (BIO13). These variables exhibited correlation coefficients predominantly below |r| = 0.7, though moderate correlation was observed between the two precipitation variables (BIO12 and BIO13, r = 0.86). Despite this correlation, both precipitation variables were retained due to their distinct ecological interpretations—BIO12 reflecting overall water availability throughout the year, while BIO13 captures seasonal moisture extremes that may critically influence tick survival during peak precipitation periods. To further assess multicollinearity among predictor variables, Variance Inflation Factor (VIF) analysis was performed. All variables, including BIO12 and BIO13, exhibited VIF values below 5 (range: 1.69–2.27), indicating low multicollinearity. Consequently, all five variables were retained for model development based on both statistical thresholds and ecological significance.

### 3.3. Environmental Niche Characterization

**Figure 4:**
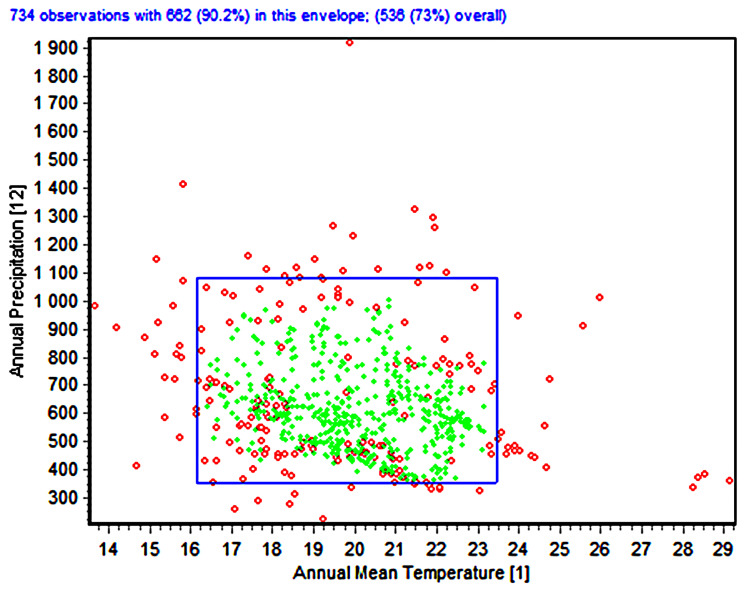
Bivariate environmental envelope analysis showing the climatic niche of *Amblyomma hebraeum* based on Annual Mean Temperature (BIO1) and Annual Precipitation (BIO12). Green points represent occurrence localities falling within the species’ environmental envelope; red points indicate locations outside the envelope either for these variables or any of the 19 variables where climatic conditions exceed the species’ tolerance limits.

The envelope test analysis indicated that *A. hebraeum* occupies a relatively specialized climatic niche within the broader environmental space available across southern Africa. Of the 734 occurrence points analyzed, 682 observations (90.2%) fell within the core environmental envelope, while the species utilized 536 unique environmental combinations, representing 73% of the available climate space within the study region (Figure 4). This pattern suggests a moderate degree of environmental selectivity, with the species concentrated in areas characterized by specific combinations of temperature and precipitation regimes.

The bivariate envelope plot for Annual Mean Temperature (BIO1) versus Annual Precipitation (BIO12) revealed the fundamental climatic requirements of *A. hebraeum*. The species’ realized niche envelope (blue rectangle) encompassed annual mean temperatures ranging from approximately 17 °C to 24 °C and annual precipitation from 350 mm to 1,100 mm. The highest concentration of presence records (green points) clustered within a narrower range of 19–23 °C and 400–800 mm, indicating optimal climatic conditions. Notably, numerous locations within the study region fell outside this envelope (red points), representing areas where climatic conditions exceed the species’ tolerance limits or where other factors preclude establishment.

### 3.4. Variable Importance and Species-Environment Relationships

**Figure 5:**
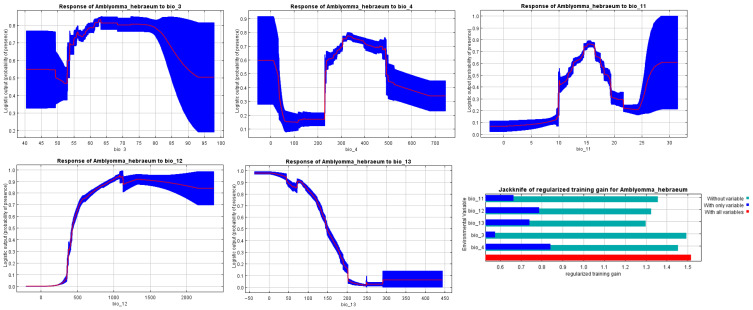
Response curves showing the relationships between the probability of the presence of a species and bioclimatic variables, and the jackknife test showing the most effective environmental variables used in this analysis.

The jackknife analysis of regularized training gain quantified the relative contribution of each environmental variable to model performance (Figure 5, lower right panel). Temperature Seasonality (BIO4) emerged as the single most important predictor, with its exclusion resulting in the greatest decrease in model performance. The dark blue bar representing BIO4 in the jackknife plot substantially exceeded those of other variables, indicating that this variable captures unique information about the species’ environmental requirements that cannot be adequately substituted by other predictors. This result underscores the critical importance of thermal stability in determining *A. hebraeum* distribution patterns.

When models were run using only individual variables (light blue bars), BIO4 again demonstrated the highest gain, confirming its strong univariate predictive power. The remaining variables showed progressively lower individual contributions in the following order: BIO11 (Mean Temperature of Coldest Quarter), BIO12 (Annual Precipitation), BIO13 (Precipitation of Wettest Month), and BIO3 (Isothermality). The full model incorporating all five variables (red bar) achieved the highest training gain, demonstrating that each variable contributes complementary information to the overall predictive framework.

The response curves revealed distinct patterns in the relationship between habitat suitability and each environmental variable (Figure 5). For Isothermality (BIO3), the species exhibited a broad tolerance range, with suitability remaining relatively high (0.7–0.8) across values from 50 to 75, followed by a sharp decline beyond 80. This pattern suggests that *A. hebraeum* tolerates moderate variation in day-to-night temperature ranges but is excluded from areas with extreme diurnal temperature fluctuations.

The response to Temperature Seasonality (BIO4) displayed a more complex non-linear relationship. Habitat suitability was highest at low seasonality values (0–150 standard deviation × 100), decreased sharply in areas with moderate seasonality (200–300), reached an intermediate plateau, and then declined again at high seasonality values (>600). This bimodal pattern indicates that the species can persist in environments with either very low or moderate thermal seasonality but is excluded from regions experiencing extreme seasonal temperature variation. This finding aligns with the species’ known preference for relatively stable thermal environments in coastal and low-elevation areas.

Mean Temperature of Coldest Quarter (BIO11) exhibited a clear threshold effect. Suitability remained minimal at temperatures below 10 °C, increased steeply between 10 and 17 °C, peaked at approximately 20 °C (suitability ≈ 0.75), and then showed a gradual decline at warmer temperatures (>25 °C). This response curve delineates both lower and upper thermal limits, with the lower threshold likely reflecting physiological constraints on overwintering survival, while the upper limit may indicate indirect effects of temperature on host availability or habitat quality.

For Annual Precipitation (BIO12), the species demonstrated a clear moisture requirement threshold. Habitat suitability was essentially zero in areas receiving less than 400 mm annually, increased rapidly between 400 and 1000 mm, and plateaued at high suitability (>0.9) for precipitation values exceeding 1000 mm. This sigmoid relationship indicates that *A. hebraeum* is largely excluded from arid regions but thrives across a wide range of mesic to humid environments, consistent with the species’ observed distribution in higher-rainfall areas of southern Africa.

The response to Precipitation of Wettest Month (BIO13) revealed a unimodal pattern with peak suitability at intermediate values (approximately 100–150 mm). Suitability declined both at very low values (<50 mm), where seasonal moisture may be insufficient to maintain humid microhabitats, and at extremely high values (>300 mm), where excessive rainfall might negatively impact tick survival through flooding of microhabitats or indirect effects on host behavior and vegetation structure.

### 3.5. Model Performance and Validation

The ecological niche model for *A. hebraeum* demonstrated excellent predictive performance across multiple evaluation metrics. The mean area under the receiver operating characteristic curve (AUC) across 10 replicate bootstrap runs was 0.94 (±0.008 SD), falling well within the “excellent” classification range (0.9–1.0). This high AUC value indicates strong discriminatory ability, with the model effectively distinguishing presence localities from background points across the environmental gradients represented in the study region.

The partial ROC analysis provided additional validation of model robustness. All 500 bootstrap iterations yielded AUC ratios significantly greater than the null expectation of 1.0 (*p* < 0.001), with a mean pROC ratio of 1.89 (range: 1.84–1.92). These results confirm that model predictions substantially exceed random expectations across the range of omission errors relevant for conservation and disease risk assessment applications. The consistency of high performance across both standard and partial ROC metrics indicates that the model captures genuine environmental associations rather than artifacts of the modeling procedure or sampling bias.

Threshold-dependent evaluation was conducted, and the mean threshold value across replicates was 0.36. At this threshold, the model achieved high performance, with sensitivity (0.866) and specificity (0.909), indicating strong discrimination between suitable and unsuitable areas. The TSS value (0.776) further confirms the robustness and reliability of the model predictions.

Variable importance analysis quantified the proportional contribution of each predictor to the final model. Temperature Seasonality (BIO4) contributed 30.2% to model fit, confirming its dominant role in determining habitat suitability. The remaining variables contributed as follows: BIO11 (23.7%), BIO12 (21.4%), BIO13 (15.9%), and BIO3 (8.8%). These contribution values align with the jackknife results, providing convergent evidence for the hierarchical importance of thermal and moisture variables in shaping *A. hebraeum* distribution.

### 3.6. Current Potential Distribution

**Figure 6:**
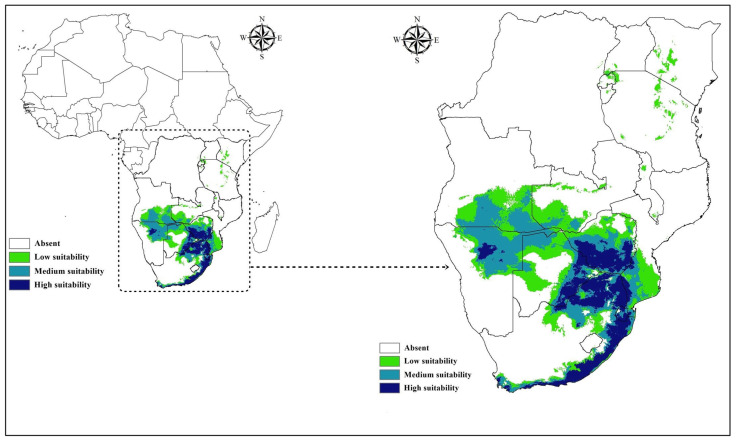
Current potential distribution of *Amblyomma hebraeum* in Africa.

The predicted current potential distribution of *A. hebraeum* exhibited strong concordance with known occurrence records and previously documented distribution patterns (Figure 6). Areas of high habitat suitability (>0.6) were concentrated along the southeastern coastal regions of South Africa, particularly the Eastern Cape and KwaZulu-Natal provinces, where warm temperatures and reliable rainfall create favorable conditions year-round. High suitability zones extended inland along river valleys and elevated areas that maintain adequate moisture despite lower overall precipitation.

Beyond South Africa, substantial areas of predicted high suitability occurred in Eswatini (Swaziland), southern Mozambique, southern and eastern Zimbabwe, and eastern Botswana. These regions share similar climatic characteristics with the core distribution areas in South Africa, featuring moderate temperature seasonality and annual precipitation typically exceeding 500 mm. Smaller patches of suitable habitat were identified in northern Namibia, particularly along the Caprivi Strip, and in Tanzania and Kenya where localized areas combine appropriate thermal and moisture conditions.

Areas of moderate suitability (0.4–0.6) formed transition zones between the core distribution and unsuitable regions, occurring in central and northern Mozambique, central Zambia, and parts of southern Tanzania. These intermediate zones may support lower-density populations or serve as marginal habitat where the species persists under locally favorable microclimatic conditions. Extensive areas across western and northern South Africa exhibited very low suitability (<0.2), corresponding to regions where high temperature seasonality, low rainfall, or temperature extremes create unsuitable conditions.

### 3.7. Limiting Factor Analysis

**Figure 7:**
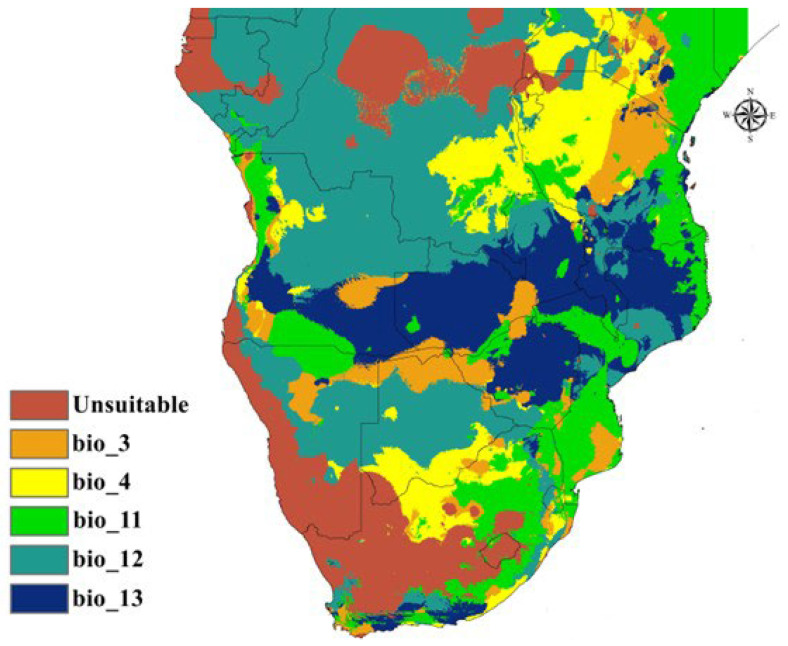
Limiting factor map of the selected variables used in modeling *Amblyomma hebraeum*.

The limiting factor analysis revealed distinct spatial patterns in the environmental constraints governing *A. hebraeum* distribution across the study region (Figure 7). Large portions of the western and southwestern study area were classified as unsuitable (value 0), indicating that environmental conditions in these regions fall outside the species’ tolerance range across multiple variables simultaneously. These areas correspond primarily to the arid and semi-arid zones of Namibia, western Botswana, and the western interior of South Africa, where a combination of low rainfall and high temperature seasonality creates inhospitable conditions.

Where suitable habitat was predicted, the dominant limiting factors showed clear geographic structuring. Temperature Seasonality (BIO4) emerged as the primary constraint in north-central and northeastern regions, appearing in scattered yellow patches throughout northern South Africa, western Mozambique, and Zimbabwe. These areas experience pronounced seasonal temperature variation that approaches or exceeds the species’ tolerance threshold, limiting distribution despite otherwise favorable moisture conditions.

Mean Temperature of Coldest Quarter (BIO11) predominantly limited distribution in coastal areas and parts of the interior plateau, shown in green throughout the map. This pattern indicates that minimum temperature thresholds during the coldest season represent a critical bottleneck for tick survival, likely reflecting physiological constraints on cold tolerance or effects on winter host availability. The spatial extent of this limitation highlights the importance of thermal buffering in determining where *A. hebraeum* can maintain viable year-round populations.

Annual Precipitation (BIO12) constituted the key limiting factor across much of the central plateau and interior regions, displayed in teal. These areas receive sufficient moisture to fall just within the species’ tolerance range but remain at the dry margin of suitable habitat. Even modest decreases in annual rainfall in these zones could render them unsuitable, making them particularly vulnerable to projected shifts in precipitation patterns under climate change scenarios.

Precipitation of Wettest Month (BIO13) restricted distribution in the southern interior and isolated patches throughout the region, indicated by dark blue zones. These locations experience seasonal moisture patterns where peak rainfall either falls short of requirements for maintaining humid microhabitats or, in some cases, exceeds optimal levels. The patchy distribution of this constraint suggests fine-scale variation in seasonal precipitation regimes that creates a mosaic of suitable and unsuitable conditions.

Isothermality (BIO3) showed limited spatial expression as a primary constraint, appearing in orange only in discrete patches. This minimal role as a limiting factor aligns with the relatively broad tolerance range observed in the response curve analysis, suggesting that extreme diurnal temperature variation represents a constraint only in specific locations rather than a widespread limitation across the species’ range.

This spatial heterogeneity in limiting factors underscores the complex interplay between thermal and moisture gradients in shaping *A. hebraeum* distribution. The analysis reveals that different environmental variables constrain the species in different portions of its range, with implications for understanding both current distribution patterns and likely responses to future environmental change. Areas where precipitation currently limits distribution may respond differently to climate change than regions constrained primarily by temperature variables, necessitating region-specific consideration in predictive modeling and risk assessment.

### 3.8. Future Distribution Projections Under Climate Change

**Figure 8:**
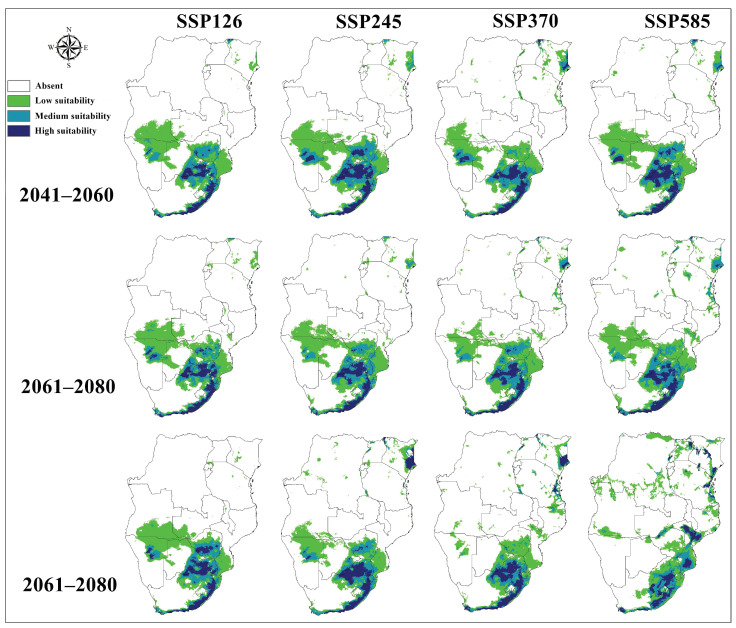
Predicted future potential distribution of *Amblyomma hebraeum* under four future shared socioeconomic pathways of climate conditions from 2041 to 2100.

**Figure 9:**
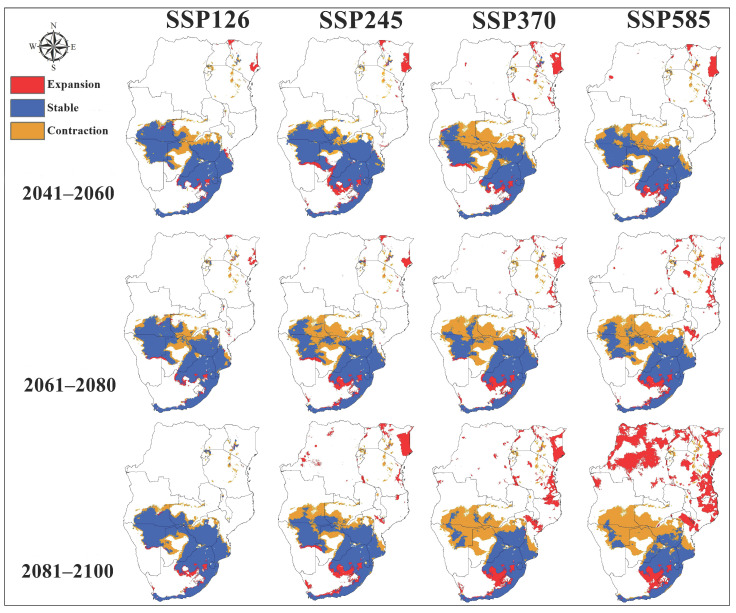
Consensus maps of predicted future climate suitability of *Amblyomma hebraeum* under four future shared socioeconomic pathways of climate conditions from 2041 to 2100 showing the gain and loss in habitat of this species.

Projections of *A. hebraeum* distribution under future climate scenarios revealed substantial anticipated changes in habitat suitability across the species’ range, with considerable variation among scenarios and time periods (Figure 8 and Figure 9). Overall, the models projected a general contraction of suitable habitat under most future scenarios, though the magnitude and spatial pattern of change differed markedly depending on the emissions pathway and time frame considered.

Transferring the calibrated models to future climate conditions showed that distributional patterns in future periods maintained broad similarities to present-day conditions, with suitable habitat remaining concentrated in eastern and southeastern regions. However, quantitative analysis of habitat area revealed consistent decreases in the total extent of suitable habitat across nearly all scenario–period combinations. The core areas of highest suitability in coastal South Africa and adjacent regions generally persisted, but marginal populations in the northern and western portions of the current range faced substantial habitat loss.

For the near-term period (2041–2060), predicted changes in suitable habitat area relative to current conditions varied considerably among emission scenarios (Figure 8). Under SSP1-2.6 (low emissions), habitat area decreased by 16.7%, while SSP2-4.5 (moderate emissions) projected a more modest decline of 10.9%. The SSP3-7.0 (high emissions) scenario indicated a 16.9% reduction, closely matching the low-emissions scenario. Interestingly, SSP5-8.5 (very high emissions) showed the smallest near-term decline at 9.3%, suggesting that initial warming may partially offset habitat loss in some cooler regions before more severe long-term impacts emerge.

By mid-century (2061–2080), the trajectories diverged more clearly among scenarios. SSP1-2.6 and SSP2-4.5 projected similar moderate declines of 11.2% and 10.7%, respectively, while SSP3-7.0 indicated accelerated habitat loss of 16.3%. Notably, SSP5-8.5 showed only a 1.5% reduction during this period, the lowest decline across all scenario–period combinations. This pattern likely reflects complex non-linear responses to warming, where temperature increases in currently cooler suitable areas initially expand thermal suitability before subsequent changes in precipitation patterns or thermal extremes drive habitat loss.

The long-term projections (2081–2100) revealed the starkest contrasts among emission scenarios. SSP3-7.0 emerged as the most pessimistic scenario with a 26.9% reduction in suitable habitat—the largest decline observed across all projections. SSP1-2.6 and SSP5-8.5 showed more moderate decreases of 12.1% and 12.0%, respectively, while SSP2-4.5 indicated the smallest long-term impact with only a 4.5% reduction. The convergence of outcomes under the lowest and highest emission scenarios by century’s end suggests threshold effects, where either mitigation prevents severe impacts or extreme warming drives habitat loss through mechanisms distinct from those operating under intermediate scenarios.

Spatially, the consensus maps across scenarios revealed consistent patterns of habitat gain and loss (Figure 9). Southern Zimbabwe and northern South Africa showed pronounced habitat contraction across most scenarios, with areas of current high suitability declining to moderate or low suitability. Eastern Botswana exhibited similar patterns of degradation. In contrast, limited areas of habitat expansion appeared in Tanzania and Kenya during later time periods, particularly under SSP5-8.5, where warming may create newly suitable thermal conditions in currently cooler highland regions.

**Figure 10:**
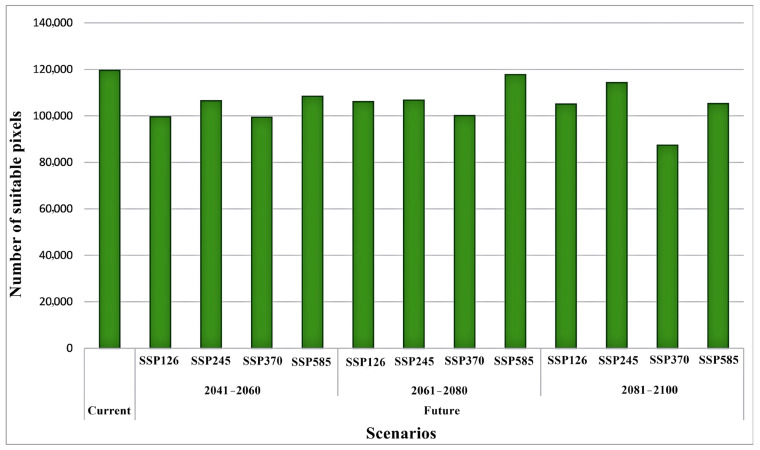
Number of suitable pixels in current and future (2041–2100) climate conditions for *Amblyomma hebraeum*.

The temporal dynamics of change revealed non-linear trajectories for several regions. Some areas showed initial habitat improvement followed by subsequent decline, while others experienced steady degradation throughout the projection period (Figure 10). The coastal regions of South Africa generally maintained high suitability across scenarios, though with some reduction in the spatial extent of optimal habitat. Interior regions proved more volatile, with dramatic shifts in suitability reflecting their position near current climate tolerance thresholds where small environmental changes produce large shifts in habitat quality.

These projections indicate that climate change will likely reshape *A. hebraeum* distribution across southern Africa, with most scenarios predicting net habitat loss and substantial geographic reorganization of suitable areas. The variation among scenarios and time periods emphasizes the importance of considering multiple climate futures when planning surveillance and control strategies for this medically and veterinary-important vector species.

### 3.9. Consensus Distribution Change Analysis

Analysis of the consensus maps across all scenario–period combinations revealed a consistent pattern of habitat contraction for *Amblyomma hebraeum*, with the magnitude and spatial configuration of change varying substantially among emission pathways and time horizons (Table 1, Figure 9). During the near-term period (2041–2060), stable habitat dominated across all scenarios, comprising 61.7–70.3% of the current suitable range. Habitat contraction was most pronounced under SSP3-7.0 (34.8%) and SSP1-2.6 (33.3%), while SSP5-8.5 showed the least contraction (23.6%) alongside the highest proportional expansion (6.1%), suggesting that initial warming may temporarily create suitable conditions in currently marginal areas under the highest-emission pathway. By mid-century (2061–2080), the stable component declined across all scenarios, with SSP3-7.0 recording the most severe contraction (48.6%) and the lowest stable area (47.3%), while SSP5-8.5 retained the greatest proportion of stable habitat (67.5%) accompanied by the highest near-term expansion (8.3%). In the long-term period (2081–2100), divergence among scenarios was most pronounced. SSP3-7.0 produced the greatest habitat loss, with stable area declining to 41.6% and contraction reaching 53.5%, representing the most pessimistic projection across all scenario–period combinations. Under SSP2-4.5, contraction similarly reached 52.0% by 2081–2100. Notably, SSP5-8.5 exhibited a markedly different trajectory, with expansion increasing dramatically to 28.7% by end-of-century—the highest expansion value across the entire projection period—reflecting a potential redistribution of suitable habitat toward currently cooler regions as temperatures increase substantially under the very high emissions pathway. Overall, the data indicate that net habitat loss is the dominant trajectory for *A. hebraeum* under most scenarios, with core stable areas concentrated in coastal South Africa (KwaZulu-Natal and Eastern Cape) and contraction most evident in the northern and interior portions of the current range.

## 4. Discussion

While previous studies have examined the distribution of *A. hebraeum*, the present study provides several important advancements. In contrast to earlier work [17,18,19] that relied on limited environmental predictors or restricted geographic extents, our study integrates high-resolution climatic data, a larger and carefully filtered occurrence dataset, and explicit treatment of multicollinearity and sampling bias. Moreover, the combined use of VIF-based variable selection, jackknife analysis, and pROC evaluation provides a more robust and interpretable modeling framework.

This study provides the first comprehensive assessment of *Amblyomma hebraeum* distribution across its entire southern African range under both current and projected future climate conditions. By integrating occurrence data from multiple host species, employing rigorous variable selection procedures, and analyzing projections from multiple climate models and emission scenarios, we have generated robust predictions of habitat suitability that carry important implications for veterinary disease management and public health planning. The predicted distribution maps show strong concordance with known occurrence records while providing new insights into the environmental factors that constrain this medically important tick species. Importantly, the detailed quantification of habitat suitability changes under multiple future climate scenarios offers new insights into the potential impacts of climate change on this medically and veterinary-important tick species.

Our results identify temperature seasonality as the primary driver of *A. hebraeum* distribution, contributing over 30% to model performance and showing the strongest relationship with habitat suitability among all tested variables. This finding aligns closely with previous studies on this species at regional scales. Tagwireyi et al. [18] similarly identified temperature seasonality as a key limiting factor in Zimbabwe, while Estrada-Peña [19] noted that *A. hebraeum* thrives in areas with relatively stable thermal regimes and struggles in regions with pronounced seasonal temperature variation. The species’ preference for low temperature seasonality reflects fundamental physiological constraints on tick survival and development, as extreme temperature fluctuations can disrupt the synchronized life cycle stages necessary for population persistence [37,38].

This pattern of temperature seasonality limitation extends beyond *A. hebraeum* to other tick species across diverse geographic contexts. Studies on *Rhipicephalus microplus* in South America documented similar constraints, with habitat suitability declining sharply in regions experiencing high seasonal temperature variation [12,63]. Likewise, modeling of *Ixodes scapularis* distribution in North America revealed that thermal seasonality acts as a primary barrier to range expansion, particularly at northern latitudes where winter temperature extremes limit overwinter survival [64]. For *Dermacentor variabilis*, Boorgula et al. [10] found that areas with moderate temperature seasonality supported higher population densities, while regions with extreme seasonal variation showed only sporadic occurrence despite seemingly adequate mean annual temperatures.

The threshold response observed for mean temperature of the coldest quarter in our study parallels findings from other tick species in temperate and subtropical regions. The sharp decline in suitability below 10 °C mirrors cold tolerance limits documented for *Amblyomma americanum* [9], where winter temperatures below this threshold significantly reduce survival of overwintering nymphs. Similarly, Springer et al. [65] demonstrated that *Ixodes ricinus* in Europe exhibits a comparable minimum temperature requirement, with populations unable to establish in areas where winter temperatures consistently fall below 8–10 °C. These convergent patterns across phylogenetically diverse tick species suggest that cold tolerance represents a fundamental constraint on tick biogeography globally.

Our analysis revealed that precipitation variables play secondary but nonetheless critical roles in defining *A. hebraeum* distribution. The sigmoid response to annual precipitation, with a clear threshold around 400 mm, effectively excludes the species from arid regions while permitting establishment across a broad range of mesic to humid environments. This moisture requirement pattern has been observed in multiple tick species across Africa. Madder et al. [66] documented similar precipitation thresholds for *Rhipicephalus appendiculatus* and *Amblyomma variegatum*, both of which require minimum annual rainfall of 400–500 mm to maintain viable populations. The importance of precipitation reflects the sensitivity of off-host tick stages to desiccation stress, as larvae and nymphs must maintain adequate water balance during extended periods in the soil and leaf litter [67].

Interestingly, our results show that extremely high precipitation during the wettest month can negatively impact habitat suitability, producing a unimodal response curve. This pattern differs from some other tick species that show monotonic increases in suitability with moisture availability. For instance, *Ixodes pacificus* in California exhibits continually increasing suitability with precipitation across the observed range [14], likely reflecting adaptation to Mediterranean climates where summer drought represents the primary limiting factor. The negative effects of excessive seasonal rainfall observed for *A. hebraeum* may result from flooding of ground-level microhabitats or indirect effects on host distribution and behavior during peak rainfall periods.

The spatial patterns of habitat change revealed by the consensus maps align with broader patterns documented for subtropical tick species facing climate change. The predominance of contraction over expansion across most scenario–period combinations for *A. hebraeum* is consistent with findings for other tick species currently operating near their thermal optima in subtropical regions [12,17]. The relatively high proportions of stable habitat in the near-term (2041–2060), particularly under SSP2-4.5 and SSP5-8.5, suggest a degree of climatic resilience in core distributional areas during the coming decades, providing a near-term window for management intervention before more severe long-term losses materialize. The contrasting behavior of SSP5-8.5 in the long-term period—showing large expansion values alongside substantially reduced stable area—likely reflects a bimodal redistribution process in which extreme warming renders currently optimal interior areas unsuitable while simultaneously opening novel climatic space in cooler highland areas of East Africa, consistent with the spatial patterns visible in Figure 9 for the 2081–2100 panel. This non-linear trajectory has been observed in other species experiencing threshold responses to temperature change [68]. The high contraction values under SSP3-7.0 across all three periods are particularly concerning from a disease management perspective: while overall suitable area diminishes, the concentration of *A. hebraeum* populations into contracting but persistent core areas may intensify local transmission of *Ehrlichia ruminantium*, potentially increasing heartwater disease burden in remaining suitable zones even as the species’ geographic range contracts—a dynamic previously documented for *Rhipicephalus appendiculatus* and East Coast fever in East Africa [66]. These findings underscore the need for scenario-specific and spatially targeted surveillance strategies that account for both the direction and magnitude of habitat change rather than relying on single-model projections.

The excellent model performance achieved in this study (AUC = 0.94, pROC ratio = 1.89) compares favorably with other recent tick distribution modeling efforts employing MaxEnt. Raghavan et al. [9] reported AUC values of 0.86–0.91 for *Amblyomma americanum* across North America, while Okely and Al-Khalaf [69] obtained an AUC of 0.92 for *Rhipicephalus annulatus*. Our slightly higher performance may reflect several methodological improvements, including the use of updated WorldClim 2.1 data [27], rigorous spatial filtering to reduce sampling bias [25], and comprehensive variable selection procedures combining correlation analysis, PCA, and jackknife testing. The integrative framework adopted in this study—combining statistical filtering, ecological reasoning, and model-based evaluation—ensures that variable selection is not driven solely by statistical artifacts but reflects meaningful environmental drivers of species distribution.

The incorporation of occurrence records from diverse host species represents a key strength of this study compared to previous work on *A. hebraeum*. Tagwireyi et al. [18] restricted their analysis to cattle-derived records, potentially underestimating the full climatic niche breadth given that the species utilizes numerous wildlife hosts across its range. Our more inclusive approach better captures the realized niche across the species’ complete ecological amplitude, similar to the multi-host approach employed by Alkishe et al. [70] for *Ixodes scapularis*, which demonstrated that single-host datasets can substantially underestimate range limits and climatic tolerances.

Our decision to exclude NDVI and other remotely sensed vegetation indices, while potentially limiting in some respects, aligns with recent recommendations for long-term climate projection studies. Climatic variables consistently outperform vegetation-derived predictors in modeling African tick distributions, primarily because climate exerts direct physiological effects while vegetation acts as an indirect proxy. Furthermore, the lack of reliable future NDVI projections under climate change scenarios makes vegetation indices problematic for temporal model transfer [71]. Studies incorporating NDVI into current distribution models but then projecting with climate variables alone introduce methodological inconsistencies that may inflate uncertainty in future predictions.

The projected habitat loss for *A. hebraeum* under future climate scenarios aligns with a growing body of evidence suggesting that many tick species in subtropical and tropical regions will experience range contractions rather than expansions as climate warms. Our projections of 4.5–26.9% habitat loss by 2081–2100 were quantified based on the number of suitable pixels and compared descriptively across scenarios, as shown in Figure 10. Depending on the emission scenario, similar findings from other studies in the Southern Hemisphere have been reported. Marques et al. [12] projected 15–25% habitat loss for *Rhipicephalus microplus* in South America under high-emission scenarios, while studies in Australia have documented similar contraction patterns for tropical tick species [72].

This pattern contrasts markedly with projections for tick species at higher latitudes, which generally show range expansions under warming scenarios. Hahn et al. [14] predicted substantial northward and elevational expansion of *Ixodes pacificus* in California, while Porter et al. [15] documented similar poleward shifts for the same species across western North America. In Europe, *Ixodes ricinus* has already exhibited range expansion into Scandinavia as winter temperatures moderate [73]. These divergent patterns reflect fundamental differences in current climate constraints: temperate species currently limited by cold temperatures benefit from warming, while subtropical species already near their thermal optima face degradation of suitable conditions.

The non-linear trajectory of change observed in our projections, where SSP5-8.5 shows minimal mid-century decline but substantial long-term loss, warrants careful interpretation. Similar temporal dynamics have been reported for other species where initial warming creates transient favorable conditions in marginal areas before subsequent precipitation changes or temperature extremes drive habitat loss [74]. For *A. hebraeum*, this pattern may reflect initial expansion into currently cooler highland areas followed by later contraction as temperature seasonality increases or precipitation patterns shift unfavorably.

The consistency of habitat loss across most scenarios and time periods suggests high confidence in the general direction of change, even as the magnitude varies. This degree of agreement among projections exceeds that reported for many temperate tick species, where model consensus remains lower [64]. The stronger signal in our results likely reflects the relatively simple climate constraints operating at subtropical latitudes—primarily a combination of thermal seasonality and moisture availability—compared to more complex interactions of temperature, precipitation, snow cover, and host phenology at higher latitudes.

The predicted contraction of *A. hebraeum* habitat carries important implications for heartwater disease risk and livestock management strategies across southern Africa. Paradoxically, habitat contraction may not translate directly to reduced disease burden. As suitable habitat shrinks, remaining populations may concentrate in core areas, potentially increasing local tick densities and transmission intensity. Similar dynamics have been observed for *Rhipicephalus appendiculatus* and East Coast fever in East Africa, where habitat loss coincided with localized disease intensification in remaining suitable areas [66].

The spatial heterogeneity revealed by our limiting factor analysis provides actionable information for targeted surveillance and control efforts. Areas where precipitation currently limits distribution represent zones of potential rapid change under shifting rainfall patterns, warranting enhanced monitoring to detect range shifts. Conversely, regions where temperature seasonality imposes primary constraints may prove more stable unless future climate scenarios produce unexpected changes in seasonal temperature variation. This spatial specificity in climate constraints enables risk managers to prioritize surveillance resources toward areas most likely to experience significant shifts in tick distribution and abundance.

The projected emergence of limited suitable habitat in Tanzania and Kenya under some scenarios deserves particular attention, as these areas currently harbor only marginal populations. If warming creates newly favorable conditions in East African highlands, heartwater disease could potentially expand into regions with naive livestock populations lacking prior exposure to *Ehrlichia ruminantium*. Similar expansion of disease risk into previously unaffected areas has been documented for other tick-borne pathogens responding to climate change, including *Anaplasma marginale* carried by *Rhipicephalus microplus* [12] and *Babesia* species vectored by *Ixodes ticks* [64].

The projected northward expansion of suitable habitats for *A. hebraeum* could have important implications for the spread of heartwater disease. As areas that were once unsuitable become more favorable, the tick may establish itself in new regions where it was previously absent. This creates a higher risk of transmitting *Ehrlichia ruminantium*, particularly among livestock populations that have not been exposed before and therefore lack immunity. Consequently, these regions may experience increased infection rates, along with higher morbidity and mortality in cattle, sheep, and goats, leading to economic losses. In addition, the introduction of the vector into new environments may challenge current control efforts, making it necessary to strengthen surveillance, improve early detection, and apply more targeted tick management strategies to reduce the disease’s impact.

While this study represents a substantial advance over previous regional assessments of *A. hebraeum* distribution, several limitations warrant acknowledgment. First, our models incorporate only climatic variables and do not explicitly account for host species distributions, which may shift independently under climate change. The realized distribution of *A. hebraeum* depends critically on the availability of suitable vertebrate hosts, and discordant responses of ticks and hosts to climate change could produce mismatches not captured in climate-only models [67]. Future modeling efforts might productively incorporate host distribution projections, though such approaches introduce additional uncertainty from host distribution models.

Second, our projections assume niche conservatism—that the species’ climatic requirements remain constant through time. Evidence from other tick species suggests that rapid evolution of thermal tolerance can occur over decadal timescales [73], potentially enabling populations to persist in areas our models predict as unsuitable. However, the direction of such evolutionary change remains difficult to predict, and assuming niche conservatism represents a reasonable null hypothesis for informing near-term management decisions.

Third, we did not explicitly model dispersal limitations or population dynamics, treating the species as capable of immediately colonizing any suitable habitat. In reality, range shifts require dispersal by hosts or other vectors, processes that may lag behind climate change by decades [74]. Our models therefore represent equilibrium distributions under different climate scenarios rather than predictions of realized distributions at specific future dates. Integrating dispersal models with climate projections, as attempted for some European tick species [73], would provide more realistic temporal predictions but requires data on dispersal rates and barriers that remain largely unavailable for *A. hebraeum*.

Fourth, the interspecific interactions were not explicitly incorporated into the modeling framework. Previous studies have shown that *A. hebraeum* may form parapatric boundaries with *A. variegatum*, indicating that biotic factors such as competition or reproductive interference may influence the realized distribution [8,75], so the interaction between the two tick species should not be ignored. Finally, our ensemble approach averages predictions across multiple GCMs but does not fully capture uncertainty in future climate projections. Different climate models show varying degrees of agreement for southern Africa, particularly regarding precipitation changes [40]. This uncertainty propagates through to habitat suitability predictions, though our use of multiple scenarios and models provides some indication of the range of possible outcomes.

## 5. Conclusions

This study provides the most comprehensive assessment to date of *Amblyomma hebraeum* distribution across its complete southern African range, employing updated climate data, rigorous variable selection, and multiple future scenarios. Our results demonstrate that temperature seasonality and precipitation patterns jointly constrain the species’ distribution, with projected climate change likely to reduce suitable habitat across most scenarios. These findings align with broader patterns observed for other tick species in subtropical regions while contrasting with expansions projected for temperate species. The spatial patterns of change identified here can inform targeted surveillance and control strategies, helping to maintain effective management of heartwater disease and other *A. hebraeum*-borne pathogens as climate continues to change. Future research integrating host distributions, dispersal dynamics, and higher-resolution climate projections will further refine our understanding of this medically and economically important vector species.

## Figures and Tables

**Table 1:** *Amblyomma hebraeum* projected habitat change (%) under four climate scenarios (SSP1-2.6, SSP2-4.5, SSP3-7.0, and SSP5-8.5) across three future time periods (2041–2060, 2061–2080, and 2081–2100). Values represent the percentage of the current suitable range classified as Expansion (newly gained suitable area), Stable (persistently suitable area), and Contraction (lost suitable area), derived from consensus ensemble maps generated from four CMIP6 Global Climate Models (BCC-CSM2-MR, CNRM-ESM2-1, MIROC6, and MIROC-ES2L).

Time Period	Climate Scenario	Expansion (%)	Stable (%)	Contraction (%)
2041–2060	SSP1-2.6	4.2	62.5	33.3
	SSP2-4.5	5.8	68.4	25.8
	SSP3-7.0	3.5	61.7	34.8
	SSP5-8.5	6.1	70.3	23.6
2061–2080	SSP1-2.6	3.8	55.9	40.3
	SSP2-4.5	4.6	56.2	39.2
	SSP3-7.0	4.1	47.3	48.6
	SSP5-8.5	8.3	67.5	24.2
2081–2100	SSP1-2.6	3.5	55.1	41.4
	SSP2-4.5	3.2	44.8	52.0
	SSP3-7.0	4.9	41.6	53.5
	SSP5-8.5	28.7	38.4	32.9

## Data Availability

All data are available through the manuscript and Supplementary Materials. The data used are available through open sources: WorldClim, VectorMap and iNaturalist and all of our work can be repeated and reproduced according to our methodology.

## Supplementary Materials

The following supporting information can be downloaded at: https://www.mdpi.com/article/10.3390/ani16101455/s1, Table S1: *Amblyomma hebraeum* primary data; Table S2: *Amblyomma hebraeum* unique records; Table S3: *Amblyomma hebream* 5 km rarefied points.
